# Keys to well-being in older adults during the COVID-19 pandemic: personality, coping and meaning

**DOI:** 10.1080/17482631.2022.2110669

**Published:** 2022-08-08

**Authors:** Martin Mau, Anne-Maj Fabricius, Søren Harnow Klausen

**Affiliations:** a Department of Psychology, University of Southern Denmark, Odense, Denmark; b Health, Social Work and Welfare Research, UCL University College, Odense, Denmark; c Health Sciences Research Centre, UCL University College, Odense, Denmark; d Department for the Study of Culture, University of Southern Denmark, Odense, Denmark

**Keywords:** well-being, older adults, COVID-19, theoretical issues in well-being research

## Abstract

**Purpose:**

During the COVID-19 pandemic, older adults were portrayed as an at-risk group. While this may have been true in some respects, empirical studies on mental health, including well-being were conflicting. Some studies found that older adults demonstrated a notable emotional resilience against the impacts of the pandemic. In this study, we qualitatively examine how older adults understand well-being and how they approached pandemic’s potential influence on their well-being.

**Methods:**

17 older adults participated in the study, out of which 14 were interviewed and three provided written responses to a set of questions.

**Results and conclusions:**

Through Interpretative Phenomenological Analysis, three themes emerged:adaptation, control, and a sense of community. We use them to discuss three central questions within well-being theory and research: How far does well-being depend on personal traits and how far does it depend on the environment? How far do people adapt to changed circumstances, and how far is such adaption conducive to maintaining genuine well-being and not just a lowering of standards of comparison? How far does subjective well-being depend on individual and momentary experiences and how far does it depend on the larger temporal and social context of an individual?

## Introduction

The COVID-19 pandemic threatened not only physical health but also well-being globally (Carstensen et al., 2020). In the public discourse, older adults were viewed as a particularly vulnerable group (Ayalon et al., 2020). While this may be true in some regards, including the risk of serious physical health complications should one become infected, the picture may be more complicated concerning psychological qualities^1^ such as well-being.

## Theoretical background

In well-being theory and research, views continue to be divided with regard to three central questions:

First, how far does wellbeing depend on character traits and fundamental attitudes of a person, and how far does it depend on external circumstances and specific, contingent factors? Much theorizing and research have been devoted to examining the latter kinds of dependency, pointing to the importance of specific social settings and relationships, societal conditions, achievements, goods, or experiences. But others have emphasized the importance of stable dispositions to respond favourably to one’s situation (Haybron, 2008), echoing views from classical philosophy that the key to maintaining wellbeing is to foster an attitude that enables one to remain unaffected by the inevitable twists and turns in life. Whereas such an approach takes the factors underlying wellbeing to be partly within one’s own—albeit indirect—control, there is also evidence that inherent personality traits, like introversion or extroversion, substantially determine one’s chances for achieving wellbeing (Lykken & Tellegen, 1996; Nettle, 2005, pp. 91ff.). Almost everybody agrees, eventually, that overall wellbeing depends on both circumstantial and personal factors, but their relative importance and the mode of their interaction remain quite controversial.

Secondly, a related question concerns how to understand the pervasive tendency of people to adapt to new circumstances and revert to a certain baseline of self-reported wellbeing (Lyubomirski, 2011; Sheldon & Lucas, 2014). Is it evidence that external circumstances matter less, and that people can do relatively little, individually or collectively, to improve their well-being (Brickman & Campbell, 1971; cf., Binder, 2010)? Does it show that people react with resignation or even self-abnegation to difficult circumstances, actually doing less well, but trying to convince themselves and others that they are still okay, perhaps because of conventional norms and expectations (Elster, 1983; Sen, 1992, 1999; Nussbaum, 2000; Haybron, 2008, p. 79ff.; Klausen, 2020)? Or does it rather show that people possess genuine positive powers of adaptation, that they are actively using individual or collective strategies and coping techniques that enable them to thrive even under seemingly adverse and worsening conditions (Schulz & Decker, 1985; Wahl, Schilling et al., 1999, 1999; Wahl, Oswald et al. 1999; Fry & Keyes. 2010; Bruckner, 2009; Schroeder, 2015; Van der Deijl, 2017; Mitchell, 2018; Klausen, 2020; Klausen et. al. 2021)?

Thirdly, opinions (and methodological choices) differ as to how far wellbeing should be considered a matter of an individual’s narrow personal state, that is, of her own present experiences and activities, and how far it should be seen as involving factors beyond this, like the overall features of her life, her sense of meaning and purpose and relations to close and distant others. Some approaches target very narrow personal states (e.g., Kahneman, 2000), while others rely on more global evaluations (Diener et al., 2002) or take into account more objective features of a person’s life and environment when assessing her wellbeing (Ryan & Deci, 2001; Sen, 1993). Until now, however; few have taken seriously the idea that present wellbeing might depend on the more extended features of a human life (but see Velleman, 1991; Dworkin, 1993, p. 205; Hurka, 2011; Klausen, 2018a, 2018b). It has, however, been noted that people from non-Western cultures exhibit more collectivist and less individualist understanding of wellbeing than people from Non-Western cultures (e.g., Lou et al., 2008), also indicating that it is an open question how widely or narrowly the locus of wellbeing should be conceived.

It should be noted that while we have studied views on, and perceptions and experiences of, well-being (see the section Analytic procedure on methodology), we are not ourselves committed to a particularly subjective view of well-being. The qualitative research design (and the special conditions created by the pandemic) has enabled us to probe different conceptions of well-being and provide nuances to the otherwise dichotomous views.

These three questions are important to wellbeing theorizing and research in general. But they are especially pertinent to the case of older adults. Older adults are widely regarded as being especially vulnerable (Clegg et al., 2013; Fried et al., 2001; Kruse, 2017). and at the same time known to be extraordinarily adept at adapting to and coping with different situations (Fry & Keyes, 2010; Klausen, 2020). They are also believed to be typically concerned with a relatively narrow range of factors, being especially dependent on their local environment and experiences and events that have immediate emotional significance to them (Löckenhoff & Carstensen, 2004; Livingstone & Isaacowitz 2021).

## The case of COVID-19

The COVID-19 pandemic caused a significant change in the living conditions of older adults, who were mostly confined to their homes and experienced a drastic reduction of their, sometimes already limited, social contacts and opportunities for recreational activity. Many European countries implemented a lockdown in order to limit the contagion of the virus, greatly changing ways of interacting through local environments. Rules and guidelines involved abstaining from in-person social contact, which can be effective in countering disease transmission but may also reduce opportunities for social contact (Berg-Weger & Morley, 2020; Van Bavel et al., 2020). Among older adults, complying with the guidelines and suspending traditional social strategies involving in-person social contact was especially important, as they were more prone to experience the potentially lethal effects of the SARS-CoV-2 (coronavirus; Berg-Weger & Morley, 2020; Samuelsson et al., 2020). Older adults were thus vulnerable; their social life and indeed life itself were at risk.

On this basis, the older adults in society would presumably be the ones to suffer the most severe emotional consequences. However, contrary to expectations and public discourse (Ayalon et al., 2020), the emotional consequences of the pandemic did not seem as severe among older adults as it was among younger people. Studies conducted early in the pandemic showed how older adults displayed notable emotional resilience. In comparison with younger people, older adults reported less negative and more positive emotions, even though they were more aware of the risks associated with the pandemic (Carstensen et al., 2020). Another recent study echoed this result finding that older adults were less concerned about the threat of COVID-19 with regards to e.g., emotional well-being when compared to younger and middle-aged adults (Klaiber et al., 2021).

Studies conducted in later stages of the pandemic called this finding into question. When considering the more long-term consequences, older adults did not seem as resistant to the impacts of the pandemic on their well-being as first expected (De Pue et al., 2021). It has been suggested that older adults may have been coping more appropriately in the early phases of the pandemic, leading to higher well-being, but this has yet to be examined more closely (Klaiber et al., 2021).

## Aims and research questions

This study examines well-being among older adults during the COVID-19 pandemic and uses the results to discuss three fundamental issues in well-being theory: i) To what extent does well-being depend on personal character or external circumstances? ii) Should the ability of older adults to adapt to difficult circumstances be seen as a genuine key to maintaining wellbeing, or is the adaptation rather a sign of resignation or self-abnegation? iii) How far does individual wellbeing of older adults depend on a their wider social and temporal context? The main research question is more open, however, as we have basically asked how far older adults have been able to maintain wellbeing during the pandemic, and, as far as they have, what have been their primary means for doing so.

Thus, we will not attempt to directly answer these three questions, but provide input and nuances to the discussions by examining the following research questions:

*How do older adults understand the nature and conditions of well-being in light of the COVID-19 pandemic? And how do they approach the pandemic’s potential influence on their well-being?*

## Materials and methods

### Participants

17 people participated in this study. Nine of them characterized themselves as “female”, the rest “male”. 14 participants were interviewed, and 3 provided a written response to the questions from the interview schedule. The mean age was 73 years, with ages ranging from 64–97. We defined this group as older adults, as old age commonly includes people around 65 years old and older (see e.g., Steger et al., 2009; Baars, 2015, 2017; for a more qualitative definition of older adults that apply equally to our sample, see Klausen, 2020). Seven were living alone, seven were living with a partner, and three were living in an institution.

### Recruitment and interviewing

18 participants were recruited through advertisement in the DaneAge internal newsletter.^2^ To ensure diversity in the interviewee group, we attempted to create an equal distribution of men and women (approx. 50/50), a variation in age (64–90), and including interviewees with different physical and mental conditions. The interviewees also included single people, people who had lost their spouse (widow/widower), people with physical limitations (e.g., due to illness, previous strokes, impaired sight or hearing), people with a history of depression, and people with no loss of functionality. Additionally, we recruited elderly interviewees from different geographical areas in Denmark which meant that all five regions in Denmark were represented. Sampling was therefore purposive, which is a recommended procedure within studies building on the IPA approach, to ensure that participants have experiences relevant to the research question (Smith, 2016). Moreover, in light of the phenomenological background of this approach, we strived to include a “reasonably homogenous sample” to allow for the examination of “convergence and divergence in some detail” (Smith et al., 2009, p. 3).

Due to health considerations in light of COVID-19, participants were excluded in case they did not have the technical capability to participate in the study using a computer or phone. Participation in the study was voluntary, and written informed consent was obtained from all individual participants prior to interviewing. Interview transcripts were stored securely, and personal details were removed from the individual transcript files. The interview schedule was developed in collaboration with colleagues from XX, on the basis of a schedule from an earlier study of older adults’ wellbeing but adapted to the COVID-19 case. It was not pilot tested also because of time constraints and the open and flexible nature of the IPA approach. It contained 22 questions (13 factual questions and 9 opinion questions). One interview was conducted via Skype, 13 as phone interviews (duration from 15.02 minutes to 50.56 minutes). All interviews were conducted in Danish, which was also participants’ native language, and were transcribed verbatim.

### Methodological design and analytic procedure

To analyse the 14 transcripts, and the three written responses from older adults living in care homes, the authors used Interpretive Phenomenological Analysis (IPA; Smith et al., 2009). In using this approach, they strived towards getting an understanding of the participants’ lived experience of well-being during the COVID-19 pandemic, with emphasis on their notions of well-being, what they believed wellbeing was conditioned upon, and how they approached possible influences on their wellbeing.

The IPA method builds on phenomenology, hermeneutics, and idiography. The phenomenological component stems from theorists such as Husserl (see e.g., Husserl, 1983) and in IPA, this implies that the researcher strives towards identifying essential qualities of the participants’ lived experiences. The interpretative, or hermeneutic, aspect builds on, among others, Heidegger (see e.g., Heidegger, 1962), and on the notion, that experience is inextricably linked with interpretation. In this sense, it is believed within IPA, that the analytic procedure is based on the researcher, trying to make sense of the participant, who is also trying to make sense of their experiences (Smith et al., 2009; known as the double hermeneutics). This implies that the researcher works actively with his/her interpretations, enabling the phenomenon in question, in this case, aspects related to wellbeing, to come forth. Finally, IPA has an idiographic component, implying that the aim is to identify particular experiences for particular people, rather than making broad generalizations. It is believed, that focussing on the particulars enables a nuanced, detailed analysis, thus providing room for the complexities associated with human psychology.

IPA was originally articulated within health research (Smith, 1996, 2016). Using it to study well-being during a COVID-19 lockdown seems suitable due to the health impacts the virus posed to participants. Moreover, in line with the idiographic component of IPA, this approach is suitable for studying areas that are new or under-researched (Woolway & Harwood, 2019). Well-being among older adults is, in itself, not new or under-researched, but researching well-being in the light of the pandemic may presumably shed light on new aspects of ageing and well-being.

All three authors conducted the first five steps outlined in Smith et al. (2009) individually: reading and re-reading transcripts, making initial notes in an exploratory fashion, developing emergent themes, creation of superordinate themes, analysing subsequent transcripts, and finally, looking for patterns between transcripts. However, as IPA is a flexible method, we adjusted the analytic procedure, to allow for a joint analytic effort, working with different interpretations from the authors until a consensus was reached. Themes were formulated through meetings and the interchanging of fragments of the analysis. The analytic procedure thus moved back and forth between steps three and six. Participants were not invited to comment on the formulation of themes, as this is not a requirement following quality evaluation of IPA-studies (Smith, 2011).

## Results

To answer the research question, the following presents the themes that emerged during the analysis. These themes focus on the nature and conditions of well-being during the COVID-19 pandemic. Moreover, they focus on how the participants approached the pandemic’s influence on their well-being. Three themes emerged; the first regarding the importance of being able to adapt to the changing circumstances; the second regarding the experience of control and the importance of transparency in guidelines and restrictions, and the third regarding a sense of community at a societal or national level and related sense of meaning and purpose.

### Theme a) adaptation

One central aspect of the participants’ well-being had to do with whether they could adapt to the changing circumstances. The lack of physical contact with grandchildren or the potential economic difficulties were some of the aspects of the lockdown, which could potentially have a large effect on their daily lives and well-being. Questions arose for the participants of how one would manage to adapt to the situation and feelings of insecurity. One participant, a woman 66 years old, explained how her husband lost his job and thus had to face a sudden decrease in income: *[…] when you shut the whole thing down, what will that mean? And the following days it was, it was more emotional, like, what will it mean for us? Also now, as my husband is retired, but he works as a school bus driver. And the day after the lockdown, he did not have a job. And you do not get anything when you are retired, because you can’t get insurance.*

In the wake of the changed circumstances, some described how they managed to adapt, cushioning the impact of the lockdown. Several participants noted the need for being creative and getting the best out of the situation, and how the pandemic had prompted them to muster abilities and resources they had previously been aware of. One participant stated that *I want to be on the sunny side. Some want to walk in the shadows. […] the positive, realist attitude works for me.*

In regards to the changed social circumstances, some participants managed to find new and rewarding ways of meeting. A participant, a man 69 years old, explained how he could only see his family to a very limited extent, meeting at a train station, standing away from each other. However, through doing what was possible, he still managed to find great joy in their meeting: *We met when society had opened up just a little bit more. Down on the platform, again. And they were on one side, while we were on the other side. And we talked and had a really good time. Unfortunately, we could not hug those two kids, but then that was it. But it was a wonderful day. That I can tell you. Even though it was for just an hour.*

### Conditions of adaptation: staying active

Not all participants, however, felt that they were able to adapt to the changed circumstances. The limitations on in-person social contact made some participants feel passive, settling into a state of resignation. This was the case among some participants who had been volunteers in organizations before the lockdown. With the abrupt end of such activities, daily life started to seem boring and trivial. One interviewee, a man 74 years old, stated: *But there has been a giant black hole there, for those three months […] You know that self-isolation, right? We were two people here, for, well actually for two months, where we did not see anybody.*

On the other hand, some experienced how the situation provided new ways of being socially engaged. Some participants felt that the lockdown had changed the social dynamics in the way they interacted with friends and family and that it was possible to use this as an advantage. One participant explained how the risk of transmission necessitated changes in otherwise ingrained ways of being together, for example, by changing the setting of their meets from formal dinners to more laid-back gatherings in playgrounds and parks. One participant, a woman aged 65, explained how she and her family had started to see more clearly the values of the relationship through this process: *[…] we are more open towards each other, and we are more straightforward in our approach to each other. There aren’t as many, there aren’t as many reservations. It is wonderful. […] And my children, my children have confirmed for me that, well … they do want me.*

Despite, and in this case because of, the limitations on how to be together, the very meaning of being together became more salient. The need to rethink social interactions provided a sense of hope and also renewed the expectations of social life, illustrating how, even though the social life among the participants was greatly reduced, some learned from it and found new and more rewarding ways of being together. As the 65-year old woman, who felt that the values of her relations had come more clearly into focus, stated: *I do not want to go back completely to how things were before*

However, not all participants felt the need to actively change former ways of interacting. For some, staying active meant merely focusing on their families’ health and wellbeing, enjoying how well one has fared in life so far. One participant shared the reflection that … *[O]f course it means a lot to you, when you reach my age, to have children and grandchildren and can enjoy thinking about them doing well and being healthy. And of course, it can cause you worries if they are not, but generally … […] Of course I know that most of life is past […] but I am happy and content*. Similarly, an 86-year-old care home resident responded to the question “what does happiness mean to you?” with invoking a collective subject of well-being, answering simply: *that my family and I are doing well.*

### Theme b) control

The participants related their well-being to a sense of control, as it was important to feel safe and calm in their daily lives. A few of the participants did feel afraid and uncertain as to how the spread of the virus would progress. One participant, a woman 66 years old, talked about the virus as an invisible enemy: *[…] it is also something about not being in control anymore, all of a sudden. That you have an enemy, which is invisible, and how do you make that work in everyday life, all of a sudden?*

However, most believed that the risk of infection had been contained. All the participants explained how they thought extensively about the virus, and continuously stayed up to date with how the situation progressed. Although the participants knew they had a heightened risk of a difficult course of illness, should they become infected, due to their age, their sense of risk remained largely abstract, and in their daily lives they felt safe. They were largely able to conduct their daily lives without nervousness or fear. One participant, a woman aged 77, explained: *I have been well. Naturally, I have told myself that it would be unfortunate to become infected, and that is also why I have been trying to stay isolated […]. So, it is not something that has taken up space in my everyday life, because I know I have been taking care of myself.*

### Conditions of control: knowing what to do

Although the virus seemed to spread unpredictably, being proactive about doing what seemed necessary according to the guidelines helped them maintain their sense of control. Upholding a sense of control and sense of safety thus depended on the messages they got, and they emphasized how the consistency of official guidelines and regulations was essential. Disagreement or discrepancies were seen as an indication of lack of knowledge, and in cases where the guidelines seemed paradoxical or where the authorities gave diverging messages, several of the participants experienced great frustration. As one participant, a man, 71, explained: *And that ruins things for me because then you can’t trust what people are saying. That’s been bothering me.*

Illogical or diverging messages would challenge the older adults’ sense of control and safety. Though the restrictions required for them to make extensive sacrifices, reducing e.g., contact with grandchildren, they believed it was a necessary and worthwhile response. One participant, a woman 66 years old, explained the need to make sacrifices: *[…] there were some limitations in our rights. But that was how we could get out of this in a good way. And […] even though I did not know it at the time, then I understood, that this is what we had to try. […] That was how it could be done. That is how I thought at the time, and that is how I still think.*

However, not all participants felt the need to do anything different from what they would usually do. One participant flatly denied that the COVID-19 situation had affected her happiness in any way, pointing to her personality and general attitude to life as the basis for her resilience: *So it must be something in my DNA. I am positive, extrovert and optimist. And that’s why I think that until now, I have gone through this period in a good way. I have been happy and content*. Another participant likewise pointed to her personality, but as a more negative or at least restraining factor: *But I think it is simply my nature […]. [W]hen I am together with other people, well, I don’t go around being negative, but I am not the type who is buoyant, if you can put it like that. And I have never been. It has nothing to do with this [the pandemic]. It’s just in my genes, I think. That’s my own view of myself. … I don’t know if you can say there is a lack of joy [in my life]. But it is just who I am. I think it’s hard to change*. A third participant, a 76-year-old married male, mentions being *somewhat of a solitary person* as a key to having remained unaffected by the pandemic, concluding: *so, well, it has just gone on very much as we also do it.*

### Theme c) a sense of community

The older adults’ daily lives during the lockdown were integrated into a societal fabric, and their well-being depended on experiencing themselves as being part of society as a whole. Some felt that they, and society more generally, had been brought more closely together by sharing and shouldering a common challenge. The participants believed that most people had been capable of transcending self-interests in order to secure the most proper response to the pandemic. A participant, a man 71 years old, explained: *There is one thing that COVID-19 has led to in society. And I actually think that it is something positive. We have become incredibly good at being united about something. […] And that is something I see very rarely among people: that everyone adjusts [to each other], right?*

Participants described how the government mirrored this tendency by acting and communicating less like a party or political fraction, and more like an authority to lean on in difficult times. One participant, a woman 77 years old, explained how she had not voted for the currently ruling party, but how she was now grateful that somebody had, as the government seemed to cooperate closely with health authorities, thus indicating how former disagreements were sidelined for the sake of a greater good. For some, party affiliation or other ideological divides thus disappeared during the lockdown: *I did not vote for the government, but I am happy that somebody did because I think they have done a good job. […] The politicians and the prime minister have said that […] we do not have all the right answers for this—we are going to make mistakes. And they undoubtedly have, but on an overall level, I think they have done a good job.*

### Conditions of community: being seen

Some contrasted the national experience with reports from other countries with different approaches to the pandemic, and in that light felt a sense of pride in seeing how well their government had managed. One participant, a woman 65 years old, explained: *It is also remarkable how different the people in power have attended to the peoples’ interests and taken care of them […] I thought it was scary as I just said, but I am also very proud of living in a country such as Denmark, where you have, where somebody has the courage to make the decisions that were made.*

However, just as the notion of one country, one people became increasingly important during the lockdown, this also created rifts within the population. Political statements of the Danish people’s response to the virus underlining the people’s ability to abstain from many activities made one participant, who did not identify as Danish, feel excluded. Statements emphasized the importance of following guidelines, in order to avoid harm to other Danish people. This participant, who had a non-Danish country of origin, a man 77 years old, explains how the notion of the Danish people affected him: *She* [the prime minister] *has said: “We Danes, we Danes, we Danes”, and excuse me, but I am not Danish. I am what I am, and there are more than 100.000 people who are not Danish, and that seems excluding not to mention that just once. Just once she could have said: thank you, to all the citizens of Denmark* […]. *She should have been more including.*

## Discussion

The three themes have both direct and indirect significance to questions about well-being. This study was conducted in the early phases of the pandemic and confirm the results of the few studies that have already been carried out, indicating that older adults have generally been less negatively affected by the COVID-19 pandemic than was initially expected, and provide insight into some of the key factors and mechanisms behind this surprising trend. The key to well-being during the pandemic appears to be a combination of personal coping skills and social framework conditions. The interviews show that older adults are in some respects better equipped to deal with life under a crisis, having already developed a lifestyle less dependent on maintaining specific social or outward-directed activities, having prior experiences with confronting and overcoming hardships to draw on and compare with, and feeling generally grateful for having made it so far in life.

But while such age-group specific skills and proclivities are part of the key to well-being, they do not seem to be sufficient. Social framework conditions are also important. Judging from the themes, this is not so much, however, about maintaining concrete social relationships. The amount of actual social contact was seriously reduced during the pandemic, and this was noticed to be a loss, but that could be dealt with as long as other social ties appeared intact or strengthened. It is more about having sufficient proof that one is being seen and thought about (both by one’s relatives and by members of one’s community and the authorities), that one’s sacrifices are being acknowledged and contribute to the well-being of one’s community, and that other members of the community also respond properly to the crisis, providing a sense of common direction.

Moreover, the themes provide evidence that (and how) adaptation can be a genuine source of well-being. The comparison with other countries, but also with conditions like being at war, or with others more seriously affected by the pandemic, helped to bring out positive aspects of the participants’ current situation, and make them conscious of, and actively engage in valuable activities. The adaptation described by the participants did not seem to entail lowering of standards, nor did it function as means for suppressing their awareness of their own predicament. Characteristically, the participants seemed well aware of the negative aspects of their situation, mostly starting out with emphasizing how the pandemic had reduced their possibilities for social contact and activities. The coping was active, and so not just *accommodative* but rather *assimilative*, as it did not consist in disengagement or lowering of aspirations, but rather in finding new ways of maintaining the same level of well-being as before (for this distinction, see, Boerner, 2004; Brandtstädter, 2015). One participant talked explicitly of “converting” her social engagement into reshaping her allotment garden.

### General conditions for well-being

As for the general understanding of well-being that emerged from the interviews and was reflected in the themes, two aspects are particularly noteworthy.

First, the participants showed implicit recognition that well-being depends on a rather subtle interplay of factors. On the one hand, they took it to be both dependent on factors not within their own present control, like how well they had mastered life in the past or which personal character or temperament they had been endowed with. On the other hand, the participants likewise emphasized the importance of personal control and prompt from one’s present social network, taking well-being to depend on active engagement and as something to be achieved.

The two kinds of factors were not, however, understood as independent or contrasting. Some participants saw their ability to take control and reach out to others and benefit from their advice as rooted in their extrovert and positive nature. How well one’s children have been doing, how good one’s relation to one’s children now is, and more generally how well one has managed to get along, are assumedly things that one *has* been at least partially responsible for. Even the overall situation of the pandemic, the threat to health, economy, and well-being were conceived not in a fatalist manner, but as depending very much on responsible action from the authorities and society at large. In short, well-being was seen as resulting from the interplay between attitude, action, and various framework conditions.

Secondly, well-being is widely understood as being a personal and emotional matter. But the participants saw the realm of personal value and emotional satisfaction as expanding far beyond the narrow field of individual or immediate concern. Contemporary gerontology depicts older adults as being primarily interested in psychological well-being, focusing particularly on current and emotionally important relationships and goals (Carstensen et al., 2003, 1999). This picture has largely been confirmed by our study, but with the important corollary that such relationships and goals are not confined to the private sphere, even though home, garden, individual hobbies, and close relatives were frequently mentioned in the interviews. The perceived well-being of significant others, in a surprisingly broad sense—including family and friends, but also the wider community and the nation in general—seemed to affect the psychological well-being of the participants quite strongly. As mentioned above, feeling reassured that the pandemic is under control and not causing undue harm was important for the older adults to feel safe and well themselves. Empathy with those more negatively affected also seemed to be a factor in personal well-being. The example of the participant who did not identify as Danish indirectly confirms the importance of having a sense of community.

The subtheme of having mastered life so far likewise shows that well-being is understood as depending on factors reaching far beyond one’s present experiences and possibilities. Participants often answered questions about well-being, intended to elicit views about their *own current* well-being, by more or less immediately taking stock of their whole life. This is significant also because dominant theoretical approaches to well-being have been mostly atomistic and atemporal (Klausen 2018a ; Klausen 2018b). Kahneman (2000) is explicitly committed to such a view. The approach to subjective well-being championed by (Diener et al., 2002) gives more emphasis to global judgements but is also not particularly sensitive to the temporal dimension as such (though it is considered by Kim-Prieto et al., 2005). Our data do not allow us to ascribe any specific conception of the temporal character of well-being to the participants. They could be interpreted as expressing the view that how well one is faring at the moment is determined by the global features of one’s life. They could also be taken to show that older adults are less interested in their present well-being and more in the overall narrative structure of their lives (Dworkin, 1993; Velleman, 1991; and so be said to have changed the subject). They could even be taken to indicate that the older adults view having mastered their life as one big accomplishment. But although it is no possible to discriminate between such interpretations with any certainty, the data do show that the older adult’s concern for personal well-being extends far beyond their narrowly personal sphere not just by involving the social context and the well-being of others, but also reaching back in time. This tendency was not explicitly linked to the COVID-19 situation in the interviews. Yet it seems related to it in two important respects. As the COVID-19 situation made the importance of others more salient, it has also brought the significance of past experiences and bonds with family and friends more sharply into focus. And taking satisfaction in thinking about one’s past life appears to be one of the special abilities that have made older adults particularly adept at coping with the otherwise difficult situation.

### Meaning and well-being

While there was little explicit talk about meaning in the interviews, all three themes also confirm the central importance of meaning to well-being. This is probably most obvious in the case of b), as the sense of control turned out to depend very much on the actions and restrictions imposed by the government appearing purposeful and coherent. But the perception of this was also closely linked to the more general sense of community and common direction (c). And adaptation (a) consisted very much of finding new ways of staying active. All this corresponds closely to standard notions of “meaning in life”. For example, Martela and Steger (2016) take meaning in life to consist of comprehension, purpose, and value. The need for comprehension and purpose is reflected in the interviews by the requirement that actions and restrictions appear logical and that one’s own sacrifices contribute to a larger, common cause. The value component is most immediately visible in the adaptation practices. The participants described how they had needed, but also been able, to find or rediscover meaning in more solitary activities like gardening or reading, or by more strongly realizing the value of family relations or belonging to a community. Our findings also tally with recent proposals for a more detailed, multifactor analysis of the notion of meaning in life. Li et al. (2020) have suggested that the perception of one’s life having value necessary for meaning (and well-being) has both an internal and external (that is, outward-directed) dimension, comprising both a sense of one’s life being valuable to oneself and a felt significance of one’s life to others, society, and the world (see, also George & Park, 2016).

### Vulnerability and resilience

The combined importance of personal skills and resilience and sensitivity to framework conditions relates to—and helps to shed light on—the issue of vulnerability. That older adults seem, almost paradoxically, both particularly vulnerable and particularly skilled at coping with life and achieving well-being, has been noted before the pandemic (Boerner, 2004; Kruse, 2017; Klausen, 2020; Carstensen, 2019; for discussion of similar paradoxes related to other vulnerable or disadvantaged groups, see, Albrecht & Devlieger, 1999; Bickenbach et al., 2013). For vulnerability signifies a particular kind of risk or sensitivity, which need not in itself prevent or detract from a person’s well-being. It is a dispositional property, and more precisely a higher-order dispositional property (author 2019). Saying that older adults are vulnerable does not entail that they are not able to do well, nor that they are straightforwardly disposed to encounter negative experiences or events. But it does mean that the well-being of older adults is dependent on maintaining a rather fragile equilibrium between their emotional proclivities and the affordances of their environment. If this equilibrium is disturbed, older adults are particularly prone to suffer a significant reduction in well-being (The COVID-19 pandemic may have revealed how other age groups, like adolescents, are similarly dependent on particular social framework conditions, and so also vulnerable, though this is outside the scope of the present study). Hypothetically, this equilibrium may have become disturbed over time, as studies conducted in the later phases of the pandemic indicated that the well-being among older adults was severely impacted (De Pue et al., 2021).

Regarding policy implications, the themes emphasized here relate both to the governmental handling of COVID-19, but may also relate more broadly to how the well-being of older people are ensured, in light of the often discontinuous character of late-life progression (Schröder-Butterfill & Marianti, 2006). The themes that we address in this article on adaptation, control, and sense of community can be important aspects of processing, for example, the end of a formal work-life, loss of a spouse, or other such changes. With regards to policy, these topics highlight the value of initiatives targeted individual behaviour *and* social inclusion: that it is important to recognize and support not only how the individual responds to upheaval, but also society aids the older person through change.

## Limitations

The present study has several important limitations. A qualitative research design generally does not allow for wider generalizations. Our study obviously cannot verify or falsify general assumptions about the nature and conditions of wellbeing. Moreover, the IPA method is focused on exploring the lived experience of participants, through an interpretative approach. Although relatively transparently described within the IPA literature, how this interpretation is conducted can be criticized for being mysterious or unclear (Brocki & Wearden, 2006). Smith (2007, p. 11) recognized this, stating: *I am left feeling there is still a gap, however: When I try to make sense of this person saying this thing, what is actually happening?* Thus, the results of this study are necessarily subjective, which may be viewed as a limitation, if viewed through a positivistic lens (Laverty, 2003). More specifically, it is likely that our sample is not completely representative. Though we selected participants to achieve maximum diversity, there may have been some selection and especially self-selection bias in the initial phases of the recruitment process. Older adults who are less able to cope with the effects of the COVID-19 pandemic may not be willing to share their experiences, or difficult to establish contact with. It is also an open question how far the perceptions and understandings of older adults adequately reflect how well they are actually doing, or what has actually been beneficial or detrimental to their wellbeing. On the other hand, a qualitative study of this sort can elicit information about wellbeing that is not unduly influenced by preconceived theoretical notions and provide insight into the interplay between different factors. Even if it should not have shown how older adults are generally able to cope with the effects of the pandemic, it does at least show how some older adults apparently do manage to cope—and so helps to identify mechanisms and processes that potentially explain the surprisingly high level of well-being among older adults during the pandemic, and which could also be adopted by or applied to other groups of older adults.

## Conclusions

This study was conducted during the lockdown following the COVID-19 pandemic, as these circumstances allowed us to gain insight into some of the supposed underlying mechanisms of well-being. In answer to our research question on the nature and conditions of well-being during the pandemic, and how older adults approached potential influences of the pandemic on their well-being, three themes emerged. These regarded, firstly, the ability to adapt, not settling into a state of resignation, but rather actively using the changed circumstances as best as possible. Secondly, a theme regarding control also emerged during the analysis. Perceived control was important to participants in order to feel safe and calm. This, however, relied on having a clear idea on how to behave, underlining the importance that official guidelines seemed coherent and consistent. Finally, a theme regarding a sense of community emerged. Feeling part of communities on a societal or national level was important to the participants but depended on having the experience that one was seen as belonging to such communities.

These findings have implications of significance to well-being theory and research. They demonstrate how a wide variety of factors (and especially their interplay) are strongly instrumental to subjective well-being, which is not only dependent on a person’s immediate personal situation or concerns, but also sensitive to the perceived state of one’s community and general life-course and achievements. It is especially significant that the participants were themselves explicitly aware of this—both of the interplay of factors, like personal character and social environment, and of the intersubjective and transtemporal nature of prudential value (that is, of their well-being). The findings also provide evidence that adaptation need not take the form of resignation and self-abnegation, but can be a potent means for maintaining or even increasing the level of well-being, and not just lowering standards or aspirations. They further help to alleviate the seeming paradox that assumedly vulnerable groups (like, in this case, older adults) turn out to be particularly apt at dealing with crises and maintain high levels of well-being. Apart from examining the representativeness of the findings, future research could investigate in more detail how self-control^3^ is related to well-being. Last but not least, the findings could motivate more in-depth studies of the locus and scope of subjective well-being, conceptually developing and empirically testing notions of a more or less collective (author 2018a) or temporally extended subject of well-being.

## Data Availability

Research data are not shared due to privacy and ethical reasons.

## Notes

1. We assume here for the sake of simplicity that well-being is a psychological state. Even if well-being is taken to comprise more than psychological factors, as some philosophical views on well-being would have it, there is still no simple connection between the health risk and the threat to well-being.

2. The present study is part of an international comparative study of older adults well-being during COVID-19.

3. The relationship between self-control and subjective well-being has been investigated already, but extant studies have mostly focused on self-control in the sense of the ability to override or change one’s inner responses and interrupt undesired behavioural tendencies (see e.g., Hofmann et al., 2014). By contrast, the kind of self-control we found to be important to older adults’ well-being during the pandemic is a more general sense of being able to master and control one’s life and not being at the mercy of external, intransparent and potentially harmful forces (Ross & Sastry, 1999).

## References

[cit0001] Albrecht, G., & Devlieger, P. (1999). The disability paradox. High quality of life against all odds. *Social Science & Medicine*, 48(8), 977–12. 10.1016/S0277-9536(98)00411-0 10390038

[cit0002] Ayalon, L., Chasteen, A., Diehl, M., Levy, B. R., Neupert, S. D., Rothermund, K., Tesch-Römer, C., & Wahl, H. W. (2020). Aging in times of the COVID-19 pandemic: Avoiding ageism and fostering intergenerational solidarity. *The Journals of Gerontology. Series B, Psychological Sciences and Social Sciences*, 76(2), e49–e52. 10.1093/geronb/gbaa051 PMC7184502 32296840

[cit0003] Baars, J. (2015). Time in late modern aging. In J. Twigg & W. Martin (Eds.), *Routledge handbook of cultural gerontology* (pp. 397–403). Routledge.

[cit0004] Baars, J. (2017). Aging: Learning to live a finite life. *The Gerontologist*, 57(5), 969–976. 10.1093/geront/gnw089 27334803

[cit0005] Bavel, J., Baicker, K., Boggio, P. S., Capraro, V., Cichocka, A., Cikara, M., Crockett, M. J., Crum, A. J., Douglas, K. M., Druckman, J. N., Drury, J., Dube, O., Ellemers, N., Finkel, E. J., Fowler, J. H., Gelfand, M., Han, S., Haslam, S. A., Jetten, J., Kitayama, S., … Willer, R. (2020). Using social and behavioural science to support COVID-19 pandemic response. *Nature human behaviour*, 4(5), 460–471. 10.1038/s41562-020-0884-z. 32355299

[cit0006] Berg-Weger, M., & Morley, J. E. (2020). Loneliness and social isolation in older adults during the covid-19 pandemic: Implications for gerontological social work. *The Journal of Nutrition, Health and Aging*, 24(5), 456–458. 10.1007/s12603-020-1366-8 PMC7156792 32346678

[cit0007] Bickenbach, J., Felder, F., & Schmitz, B. (Eds.). (2013). *Disability and the good human life*. Cambridge University Press. 10.1017/CBO9781139225632

[cit0008] Binder, M. (2010). *Elements of an evolutionary theory of welfare: Assessing welfare when preferences change*. Routledge. 10.4324/9780203849552

[cit0009] Boerner, K. (2004). Adaptation to disability among middle-aged and older adults: The role of assimilative and accommodative coping. *The Journals of Gerontology. Series B, Psychological Sciences and Social Sciences*, 59(1), 35–42. 10.1093/geronb/59.1.P35 PMC3182571 14722337

[cit0010] Brandtstädter, J. (2015). Adaptive resources of the aging self: Assimilative and accommodative modes of coping. In N. A. Pachana (Ed.), *Encyclopedia of geropsychology* (pp. 1–8). Springer Singapore.

[cit0011] Brickman, P., & Campbell, D. T. (1971). Hedonic relativism and planning the good society. In M. H. Apley (Ed.), *Adaptation level theory: A symposium* (pp. 287–302). Academic Press.

[cit0012] Brocki, J. M., & Wearden, A. J. (2006). A critical evaluation of the use of interpretative phenomenological analysis (IPA) in health psychology. *Psychology and Health*, 21(1), 87–108. 10.1080/14768320500230185

[cit0013] Bruckner, D. (2009). In defense of adaptive preferences. *Philosophical Studies: An International Journal for Philosophy in the Analytic Tradition*, 142(3), 307–324. 10.1007/s11098-007-9188-7

[cit0014] Carstensen, L. L. (2019). Integrating cognitive and emotion paradigms to address the paradox of aging. *Cognition and Emotion*, 33(1), 119–125. 10.1080/02699931.2018.1543181 30394173 PMC6476329

[cit0015] Carstensen, L. L., Fung, H. H., & Charles, S. T. (2003). Socioemotional selectivity theory and the regulation of emotion in the second half of life. *Motivation and Emotion*, 27(2), 103–123. 10.1023/A:1024569803230

[cit0016] Carstensen, L. L., Isaacowitz, D. M., & Charles, S. T. (1999). Taking time seriously: A theory of socioemotional selectivity. *American Psychologist*, 54(3), 165–181. 10.1037/0003-066X.54.3.165 10199217

[cit0017] Carstensen, L. L., Shavit, Y. Z., & Barnes, J. T. (2020). Age advantages in emotional experience persist even under threat from the COVID-19 pandemic. *Psychological Science*, 31(11), 1374 1385. 10.1177/0956797620967261 33104409 PMC13171095

[cit0018] Clegg, A., Young, J., Iliffe, S., Rikkert, M. O., & Rockwood, K. (2013). Frailty in older adults. *The Lancet*, 381(9868), 752–762. 10.1016/S0140-6736(12)62167-9 PMC4098658 23395245

[cit0019] De Pue, S., Gillebert, C., Dierckx, E., Vanderhasselt, M., De Raedt, R., & Van den Bussche, E. (2021). The impact of the COVID-19 pandemic on wellbeing and cognitive functioning of older adults. *Scientific reports*, 11(1). doi:10.1038/s41598-021-84127-7 PMC7907111 33633303

[cit0020] Diener, E., Oishi, S., & Lucas, R. E. (2002). Subjective well-being: The science of happiness and life satisfaction. In C. R. Snyder & S. J. Lopez (Eds.), *Handbook of positive psychology. Oxford and New York* (pp. 63–73). Oxford University Press.

[cit0021] Dworkin, R. (1993). *Life’s dominion*. Vintage.

[cit0022] Elster, J. (1983). *Sour grapes: Studies in the subversion of rationality*. Cambridge University Press. 10.1017/CBO9781139171694

[cit0023] Fried, L. P., Tangen, C. M., Walston, J., Newman, A. B., Hirsch, C., Gottdiener, J., Seeman, T., Tracy, R., Kop, W. J., Burke, G., McBurnie, M. A., & Cardiovascular Health Study Collaborative Research Group (2001). Frailty in older adults: evidence for a phenotype. *The journals of gerontology*. Series A, Biological sciences and medical sciences, *56*(3), M146–M156. 10.1093/gerona/56.3.m146 11253156

[cit0024] Fry, P., & Keyes, C. (Eds.). (2010). *New frontiers in resilient aging: Life-strengths and well-being in late life*. Cambridge University Press. 10.1017/CBO9780511763151

[cit0025] George, L. S., & Park, C. L. (2016). Meaning in life as comprehension, purpose, and mattering: Toward integration and new research questions. *Review of General Psychology*, 3(20), 205–220. 10.1037/gpr0000077

[cit0026] Haybron, D. (2008). *The pursuit of unhappiness. The elusive psychology of well-being*. Oxford University Press. 10.1111/j.1467-9213.2010.659_7.x

[cit0027] Heidegger, M. 1962. *Being and Time*. (transl. J. Macquarrie & E. S. Robinson). Harper:

[cit0028] Hofmann, W., Luhmann, M., Fisher, R. R., Vohs, K. D., & Baumeister, R. F. (2014). Trait Self-Control and Well-Being. *Journal of Personality*, 82(4), 265–277. 10.1111/jopy.12050 23750741

[cit0029] Hurka, T. (2011). *The best things in life*. Oxford University Press.

[cit0030] Husserl, E. (1983). Ideas pertaining to a pure phenomenology and to a phenomenological philosophy. 1: General introduction to a pure phenomenology. Transl. F. Kersten. Kluwer.

[cit0031] Kahneman, D. (2000). Experienced utility and happiness: A moment-based approach. In D. Kahneman & A. Tversky (Eds.), *Choices, values and frames* (pp. 673–692). Cambridge University Press.

[cit0032] Kim-Prieto, C., Diener, E., Tamir, M., Scollon, C., & Diener, M. (2005). Integrating the diverse definitions of happiness: A time-sequential framework of subjective well-being. *Journal of Happiness Studies*, 6(3), 261–300. 10.1007/s10902-005-7226-8

[cit0033] Klaiber, P., Wen, J. H., DeLongis, A., & Sin, N. L. (2021). The ups and downs of daily life during COVID-19: Age differences in affect, stress, and positive events. *The Journals of Gerontology. Series B, Psychological Sciences and Social Sciences*, 76(2), e30–e37. 10.1093/geronb/gbaa096 32674138 PMC7454856

[cit0034] Klausen, S. H. (2018a). Ethics, knowledge, and a procedural approach to wellbeing. *Inquiry*, 1–17. 10.1080/0020174X.2018.1529619

[cit0035] Klausen, S. H. (2018b). Having or being? Happiness and the structure of human life. *Kultura - Media - Teologia*, 35(4), 24–38 https://kmt.uksw.edu.pl/media/pdf/kmt_35_02_Klausen.pdf.

[cit0036] Klausen, S. H. (2020). Understanding older adults’ wellbeing from a philosophical perspective. *Journal of Happiness Studies*, 21(7), 2629–2648. 10.1007/s10902-019-00197-5

[cit0037] Klausen, S. H., Christiansen, R., Emiliussen, J., Hasandedic-Dapo, L., & Engelsen, S. (2021). The many faces of hedonic adaptation. *Philosophical Psychology*, 34(7), 938–961. 10.1080/09515089.2021.1931670 34556899 PMC8452141

[cit0038] Kruse, A. (2017). *Lebensphase hohes alter. Verletzlichkeit und Reife*. Springer. 10.1007/978-3-662-50415-4

[cit0039] Laverty, S. M. (2003). Hermeneutic phenomenology and phenomenology: A comparison of historical and methodological considerations. *International Journal of Qualitative Methods*, 2(3), 21–35. 10.1177/160940690300200303

[cit0040] Li, Z., Liu, Y., Peng, K., Hicks, J. A., & Gou, X. (2021). Developing a quadripartite existential meaning scale and exploring the internal structure of meaning in life. *Journal of Happiness Studies*: An Interdisciplinary Forum on Subjective Well-Being, 22(2), 887–905. 10.1007/s10902-020-00256-2.

[cit0041] Livingstone, K. M., & Isaacowitz, D. M. (2021). Age and emotion regulation in daily life: Frequency, strategies, tactics, and effectiveness. *Emotion*, 21(1), 39–51. 10.1037/emo0000672 31478723 PMC7050390

[cit0042] Löckenhoff, C. E., & Carstensen, L. L. (2004). Socioemotional selectivity theory, aging, and health: The increasingly delicate balance between regulating emotions and making tough choices. *Journal of Personality*, 72(6), 1395–1424. 10.1111/j.1467-6494.2004.00301.x 15509287

[cit0043] Lou, V. W. Q., Chi, I., & Mjelde-Mossey, L. A. (2008). Development and validation of a life satisfaction scale for Chinese elders. *International Journal of Ageing and Human Development*, 67(2), 149–170. 10.2190/AG.67.2.c 20063848

[cit0044] Lykken, D., & Tellegen, A. (1996). Happiness is a stochastic phenomenon. *Psychological Science*, 7(3), 186–189. 10.1111/j.1467-9280.1996.tb00355.x

[cit0045] Lyubomirski, S. (2011). Hedonic adaptation to positive and negative experiences. In S. Folkman (Ed.), *The oxford handbook of stress, health and coping* (pp. 200–224). Oxford University Press.

[cit0046] Martela, F., & Steger, M. F. (2016). The three meanings of meaning in life: Distinguishing coherence, purpose, and significance. *The Journal of Positive Psychology*, 11(5), 531–545. 10.1080/17439760.2015.1137623

[cit0047] Mitchell, P. (2018). Adaptive preferences, adapted preferences”. *Mind*, 127(508), 1003–1025. 10.1093/mind/fzy020

[cit0048] Nettle, D. (2005). Happiness. The science behind your smile. Oxford University Press.

[cit0049] Nussbaum, M. (2000). *Women and human development*. Cambridge University Press. 10.1017/CBO9780511841286

[cit0050] Ross, C., & Sastry, J. (1999). The sense of personal control: Social-structural causes and emotional consequences. In A. C. S & P. J.c (Eds.), *Handbook of the sociology of mental* (pp. 369–394). Kluwer Academic/Plenum.

[cit0051] Ryan, R. M., & Deci, E. L. (2001). On happiness and human potentials: A review of research on hedonic and eudaimonic well-being. *Annual Review of Psychology*, 52(1), 141–166. 10.1146/annurev.psych.52.1.141 11148302

[cit0052] Samuelsson, K., Barthel, S., Colding, J., Macassa, G., & Giusti, M. (2020). Urban nature as a source of resilience during social distancing amidst the coronavirus pandemic. 10.31219/osf.io/3wx5a

[cit0053] Schröder-Butterfill, E., & Marianti, R. (2006). A framework for understanding old-age vulnerabilities. *Ageing & Society*, 26(1), 9–35. 10.1017/S0144686X05004423 23750062 PMC3672844

[cit0054] Schroeder, S. A. (2015). Health, disability, and well-being. In G. Fletcher (Ed.), *The Routledge handbook of philosophy of well-being* (pp. 221–232). Routledge.

[cit0055] Schulz, R., & Decker, S. (1985). Long-term adjustment to physical disability: The role of social support, perceived control, and self-blame. *Journal of Personality and Social Psychology*, 48(5), 1162–1172. 10.1037/0022-3514.48.5.1162 3158730

[cit0056] Sen, A. (1992). *Inequality reexamined*. Oxford University Press.

[cit0057] Sen, A. (1993). Capability and well-being. In Nussbaum & Sen (Eds.), *The quality of life* (pp. 30–53). Clarendon Press. 10.1093/0198287976.003.0003

[cit0058] Sen, A. (1999). *Development as freedom*. Oxford University Press.

[cit0059] Sheldon, K. M., & Lucas, R. E. (eds.). (2014). *Stability of happiness. Theories and evidence on whether happiness can change*. Elsevier.

[cit0060] Smith, J. A. (1996). Beyond the divide between cognition and discourse: Using interpretative phenomenological analysis in health psychology. *Psychology & Health*, 11(2), 261–271. 10.1080/08870449608400256

[cit0061] Smith, J. A. (2007). Hermeneutics, human sciences and health: Linking theory and practice. *International Journal of Qualitative Studies on Health and Well-being*, 2(1), 3–11. 10.1080/17482620601016120

[cit0062] Smith, J. A. (2011). Evaluating the contribution of interpretative phenomenological analysis. *Health Psychology Review*, 5(1), 9–27. 10.1080/17437199.2010.510659

[cit0063] Smith, J. A. (2016). Interpretative phenomenological analysis in sport and exercise: Getting at experience. In B. Smith & A. C. Sparkes (Eds.), *Routledge handbook of qualitative research in sport and exercise* (pp. 219–229). Routledge.

[cit0064] Smith, J. A., Flowers, P., & Larkin, M. (2009). *Interpretive phenomenological analysis. Theory, method and research*. Sage.

[cit0065] Steger, M. F., Oishi, S., & Kashdan, T. B. (2009). Meaning in life across the life span: Levels and correlates of meaning in life from emerging adulthood to older adulthood. *The Journal of Positive Psychology*, 4(1), 43–52. 10.1080/17439760802303127

[cit0066] Van der Deijl, W. (2017). Which problem of adaptation? *Utilitas*, 29(4), 1–19. 10.1017/S0953820816000431

[cit0067] Velleman, D. (1991). Wellbeing and Time. *Pacific Philosophical Quarterly*, 72(1), 48–77. 10.1111/j.1468-0114.1991.tb00410.x

[cit0068] Wahl, H.-W., Oswald, F., & Zimprich, D. (1999). Everyday competence in visually impaired older adults: A case for person–environment perspectives. *The Gerontologist*, 39(2), 140–149. 10.1093/geront/39.2.140 10224710

[cit0069] Wahl, H.-W., Schilling, O., Oswald, F., & Heyl, V. (1999). Psychosocial consequences of age-related visual impairment: Comparison with mobility-impaired older adults and long-term outcome. *Journal of Gerontology: Psychological Sciences*, 54B(5), 304–316. 10.1093/geronb/54B.5.P304 10542823

[cit0070] Woolway, T., & Harwood, C. G. (2019). Gatekeepers’ experiences of hiring a sport psychologist: A phenomenological study. *Journal of Applied Sport Psychology*, 31(4), 474–493. 10.1080/10413200.2018.1484394

